# In Vitro Evaluation of Mechanical Properties of Cention N and Its Comparison with Resin Modified Glass Ionomer Cement (RMGIC) Restorative Material as Used in Primary Teeth

**DOI:** 10.1155/2024/9420336

**Published:** 2024-01-02

**Authors:** Deepika Pai, P. A. Anirudhmaadhava, Kishore Ginjupalli

**Affiliations:** ^1^Department of Pedodontics & Preventive Dentistry, Manipal College of Dental Sciences, Manipal Academy of Higher Education, Manipal 576104, India; ^2^Kerala Institute of Medical Sciences, Trivandrum 695029, India; ^3^Department of Dental Materials, Manipal College of Dental Sciences, Manipal Academy of Higher Education, Manipal 576104, India

## Abstract

**Methods:**

22 specimens prepared with Cention and RMGIC were embedded in primary teeth mounted in acrylic for analysing shear bond strength. Shear bond strength was analysed using a universal testing machine. The modes of failure in samples were observed under a stereomicroscope and scanning electron microscope. 22 customised samples of Cention N and RMGIC were prepared and categorised as group A and group B, respectively. The flexural and compressive strengths of these samples were evaluated using a universal testing machine.

**Results:**

The shear bond strength of RMGIC was higher than that of Cention N, whereas the compressive and flexural strengths of Cention N were significantly higher than those of RMGIC. The modes of failure were predominantly adhesive followed by mixed failures.

**Conclusion:**

The results of this study suggest that Cention N demonstrated superior mechanical properties compared with RMGIC and can therefore be recommended for restorations in primary posterior teeth. Cention N being a smart, esthetic, self-cured, or dual-cured material with better mechanical properties offers a wide range of applicability in primary teeth.

## 1. Introduction

In the era of minimal invasive dentistry, adhesive restorative materials are widely used for restoration of occlusal, approximal, cervical, or multisurface cavities and as bulk restorative materials [1]. These include conventional glass ionomers, modified glass ionomers, compomers, and composites. Good compressive strength, flexural and tensile strength, high wear resistance, low water sorption, no microleakage, and biocompatibility are some of the properties that an ideal restorative material should possess [2]. Among these materials, resin modified glass ionomers (RMGIC) and composites are considered superior to others because of their better mechanical properties. Additionally, RMGIC and composites have fluoride-releasing ability that is anticariogenic.

Cention N is a new alkasite material in the class of adhesive restorative materials. It contains an alkaline filler that releases acid-neutralizing ions under acidic conditions and helps in regulating the pH, thereby preventing dental caries [3, 4]. It also releases fluoride and calcium which can remineralize incipient enamel lesions [4]. The monomer matrix of this new bioactive material consists of a mixture of urethane dimethacrylates UDMA, DCP, an aromatic aliphatic-UDMA, and PEG-400 DMA. During polymerization, the cross linking between these monomer matrix complexes imparts strong mechanical properties. The inorganic filler barium-aluminum-silicate glass imparts strength to the material [5, 6]. Manufacturers claim Cention N to be a smart material with good mechanical properties and as a competitor to existing time-tested esthetic adhesive materials such as composites and RMGIC [4, 5].

Several studies evaluated properties of Cention N-like compressive strength, flexural strength, and fluoride releasing ability, etc. [5, 7–10]. These in vitro studies demonstrate that Cention N has superior mechanical properties than GIC but not composites. Since Cention N does not offer the shade range in colour as composites, composites clearly dominate over Cention.

Since Cention N can be bulk filled and bulk cured like RMGIC, it is obvious to compare Cention N with RMGIC. Therefore, we conducted this in vitro study to evaluate and compare the compressive strength, flexural strength, and shear bond strength of Cention N on primary teeth as compared to RMGIC. The compressive strength, flexural strength, and shear bond strength of a given material can directly be correlated to its survival rate in the oral environment; hence, we evaluated these parameters [11, 12].

We hypothesised that there will be no difference in compressive strength, flexural strength, and shear bond strength between Cention N and RMGIC.

## 2. Materials and Methodology

With the approval from the institutional ethics committee (IEC no. 829/2019), Kasturba Hospital, Manipal, this in vitro experimental study was conducted. Based on the statistical assessment, a sample size of 66 was derived, 22 samples per group with a power of 80% and 95% confidence levels.

22 healthy primary teeth extracted due to preshedding mobility were used to test the shear bond strength of both the materials. 22 customised bar-shaped split moulds of 25 × 2 × 2 mm dimension made up of Vitremer TM and Cention N were used to test the flexural strength, and 22 customised cylindrical moulds of 6 mm height and 4 mm diameter made up of Vitremer TM and Cention N were used to test the compressive strength.

Cention N (Ivoclar Vivadent AG Liechtenstein), Ivoclar India, was used in the experimental group.

VitremerTM (3M ESPE), United States, is a resin modified glass ionomer cement used as a comparative material to the test in this study, along with Vitremer Primer, Vitrebond copolymer, HEMA, ethanol, and photoinitiators (3M ESPE).

### 2.1. Assessment of Shear Bond Strength

#### 2.1.1. Preparation of Acrylic Blocks

Wax blocks of dimensions 35 mm height, 15 mm width, and 15 mm thickness were carved from modelling wax. Elastomeric impression was made that served as a mould for the fabrication of acrylic blocks using cold cure methyl methacrylate resin (MMA). The prepared blocks were polished with carbide polishing paper. The 22 prepared blocks were then randomly divided into two groups of 11 blocks each, namely, in group A, 11 blocks for CN and, in group B, 11 blocks for RMGIC.

#### 2.1.2. Mounting and Preparation of the Tooth Samples

Healthy human primary teeth extracted due to preshedding mobility were cleaned with normal saline and stored for no longer than three months in distilled water before testing. A window was made in the acrylic blocks, and the teeth were placed in them. The rest of the window was filled with cold cure MMA that had been freshly mixed. To achieve a consistent flat surface, a slow-speed handpiece and a diamond disc were utilised to flatten the occlusal surfaces of the teeth. The flattened occlusal surfaces were kept exposed.

A polypropylene straw of height 4 mm and diameter 3 mm was used as a mould to build the cylinders of restorative material on the prepared tooth surface for SBS testing. Following 24 hours of storage in distilled water, the samples were subjected to testing.

#### 2.1.3. Group A: CN

Prior to the placement of the material, the tooth surface was air-dried for 60 s. CN was mixed in the powder liquid (P/L) ratio of 4.6 : 1 (by weight) using an electronic weighing scale (EWS) as per the manufacturer's directions and condensed onto the prepared tooth surface using the polypropylene straw. A plastic spatula and condenser were used to condense the material into the straw. It was then light cured for 40 s, and once set, the blocks were aged in distilled water for 24 hours.

#### 2.1.4. Group B: RMGIC

Prior to the placement of the material, the tooth surface was air-dried for 60 s. The Vitremer primer was applied onto the tooth surface with the help of an applicator tip and light cured for 20 s as per the manufacturer's instructions. Vitremer was mixed in a P/L ratio of 2.5 : 1 (by weight) using an EWS according to the manufacturer's instructions and was condensed onto the prepared tooth surface using the polypropylene straw. A plastic spatula and condenser were used to condense the material into the straw. It was then light cured for 40 s, and once set, the blocks were aged in distilled water for 24 hours.

#### 2.1.5. Shear Bond Strength (SBS) Testing

The prepared specimens were placed on the lower platform of the UTM (Instron 3366, UK) and held in place using a specifically built jig for SBS testing. A chisel-shaped debonding blade was then attached to the UTM's crosshead. The load was applied at the interface between the bonded materials at a crosshead speed of 0.5 mm/min until debonding occurred. The highest load measured during the test was divided by the bond area, and the result was expressed in MPa. These data were then statistically analysed [13].

The fractured test specimens were then studied under a stereomicroscope at magnifications of 20x, 25x, and 32x, and the failures observed were categorised as follows: (a) Cohesive failure occurs when the failure occurs within the restorative material. (b) Adhesive failure occurs when the bond between the tooth surface and the restorative material fails. (c) Mixed failure occurs when there is evidence of both adhesive and cohesive fractures. Three representative samples in each group selected randomly underwent gold sputtering and were examined under a scanning electron microscope at magnifications of 500x and 1000x. The images were analysed in order to determine the characteristics of the interface between the two bonding surfaces at the failure site.

### 2.2. Assessment of Flexural Strength

A split mould with two sections held together by two screws was fabricated with an inert material that would not react with any of the components of materials used in the study. The dimensions of the bar-shaped split mould were 25 mm × 2 mm × 2 mm.

#### 2.2.1. Group A: CN

CN was mixed according to the manufacturer's recommendations in the P/L ratio of 4.6 : 1 (by weight) using an EWS. The mix was introduced into the mould with the help of a plastic spatula. A lacron carver was used to remove the surplus material. A celluloid strip was placed over the material, and a glass slab was used to apply pressure and compact the material into the bar-shaped mould to reduce porosities. Following light curing for 40 s, the material was left undisturbed for 15 minutes with weights placed over it. After the material had set, the mould was split to retrieve the sample. The bars were aged in distilled water for 24 hours till the FS was tested.

#### 2.2.2. Group B: RMGIC

Vitremer was mixed according to the manufacturer's recommendations in the P/L ratio of 2.5 : 1 (by weight) using an EWS. The samples of Vitremer were prepared in the fabricated mould as described above for group A. The bars were aged in distilled water for 24 hours till the FS testing was performed.

#### 2.2.3. Flexural Strength (FS) Test

The prepared bar specimens were placed on the UTM (Instron 3366, UK) and kept firmly in place using a specifically developed jig for this purpose during FS testing. The crosshead of the UTM was then fitted with a chisel-shaped blade. The blade was perpendicular to the bar and made contact with it at the midpoint. Thereafter, they were loaded at a rate of 0.5 mm/min until they fractured. The maximum load recorded prior to failure, divided by the sample area, yielded FS in MPa. The data were then subjected to statistical analysis [5].

### 2.3. Assessment of Compressive Strength

For the testing of CS, customised cylindrical split moulds of dimensions 6 mm height and 4 mm diameter were fabricated with a nonreactive material.

#### 2.3.1. Group A: CN

CN was mixed as per the manufacturer's directions in the P/L ratio of 4.6 : 1 (by weight). The mix was introduced into the mould with the help of a plastic spatula. A lacron carver was used to remove the surplus material, and a condenser was used to compact the material into the cylindrical mould. Celluloid strips were placed above and below the mould to contain the material within the mould and get a smooth finish. A glass slab was used to apply pressure and compact the material into the bar-shaped mould to reduce porosities. Following light curing for 40 s, the material was left undisturbed for 15 minutes with weights placed over it. After the material had set, the mould was split to retrieve the sample and was checked for porosities. If present, the sample was discarded. The cylinders were aged in distilled water for 24 hours till the CS testing was performed.

#### 2.3.2. Group B: RMGIC

Vitremer was mixed as per the directions provided by the manufacturer in the P/L ratio of 2.5 : 1 (by weight). Cylindrical samples of Vitremer were prepared in the fabricated mould as described above for group A. The cylinders were aged in distilled water for 24 hours till the CS testing was performed.

#### 2.3.3. Compressive Strength (CS) Test

The prepared cylindrical specimens were placed on the UTM (Instron 3366 UK) which was attached to a load measuring cell and continuously recorded the load applied to the samples at a crosshead speed of 0.5 mm/min until the samples cracked. The CS was measured in MPa, and the data were analysed using statistical methods [9, 13].

### 2.4. Statistical Analysis

SPSS 22.0 (SPSS Inc., Chicago, IL) software was used to interpret the data. The level of significance was set at *p* < 0.05. The mean and standard deviation of the groups were calculated using descriptive statistics. The *Shapiro–Wilk test* was used to determine the data's normality. As the data did not follow a normal distribution, inferential statistics were used with the help of the Mann–Whitney *U* test to determine the difference between the groups.

## 3. Results

The SBS of samples in group A-CN measured the highest value of 15.77 MPa, with a mean of 7.894 MPa and a standard deviation (SD) of 4.76. The SBS of samples in group B-RMGIC measured the highest value of 28.06 MPa, with a mean of 18.89 MPa and an SD of 5.82.

**Figure 1:**
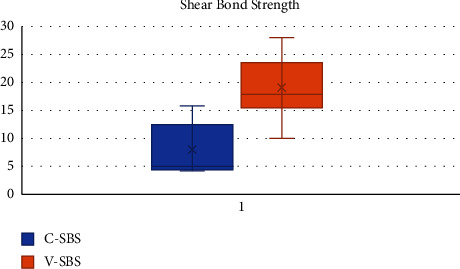
Box plot showing the comparison of shear bond strength of groups A and B expressed in MPa performed using the Mann–Whitney-*U* test. ^*∗*^*P* < 0.05 is statistically significant. The difference in shear bond strength attained between the two groups was statistically significant (*p* < 0.0008), where group B shows higher shear bond strength than group A (18.89 > 7.89).

The intergroup comparison of SBS was performed using SPSS 22.0 (SPSS Inc., Chicago, IL) software with the level of significance set at *p* < 0.05. The Mann–Whitney-*U* test applied for the two test groups reported a statistically significant difference (*p* < 0.05), wherein group B showed higher SBS than group A (18.89 > 7.9). The intergroup comparison of SBS showed that samples in group B demonstrated significantly higher SBS than those in group A (Table 1 and Figure 1).

The fracture patterns of group A and B samples were categorised as adhesive, cohesive, and mixed failures using a stereomicroscope at 20x, 25x, and 32x magnifications. In group A (CN), 9 samples showed adhesive failure and 2 samples showed mixed failure. In group B (RMGIC), 8 samples showed adhesive failure, 1 sample showed cohesive failure, and 2 samples showed mixed failure.

The SEM images of representative samples of group A (CN) showed the adhesive and mixed failures. The group B (RMGIC) showed adhesive failure, cohesive failure, and mixed failure.

The FS of samples in group A-CN recorded a maximum of 170.05 MPa, with a mean of 156.46 MPa and SD of 16.01. The FS of samples in group B-RMGIC reached a maximum of 126.02 MPa, with a mean of 90.69 MPa and SD of 15.57.

**Figure 2:**
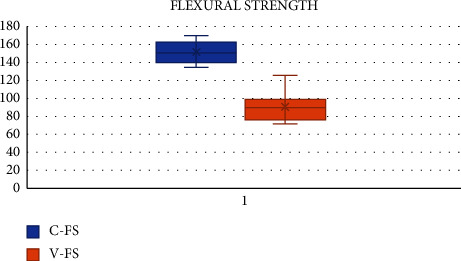
Box plot showing comparison of flexural strength of groups A and B expressed in MPa performed using the Mann–Whitney-*U* test showed a statistically significant difference (*p* < 0.05), where group A shows higher flexural strength than group B (152.11 > 90.69).

The intergroup comparison of FS showed that FS of group A samples was significantly higher than group B (*p* < 0.05). The level of significance was set at *p* < 0.05. The Mann–Whitney-*U* test performed reported a statistically significant difference (*p* < 0.05), wherein group A shows better FS than group B (156.46 > 90.69) (Table 1 and Figure 2).

The CS of samples in group A-CN reached a maximum of 251.41 MPa, with a mean of 180.84 MPa and SD of 40.84. The CS of samples in group B-RMGIC reached a maximum of 122.79 MPa, with a mean of 90.96 MPa and SD of 14.56.

**Figure 3:**
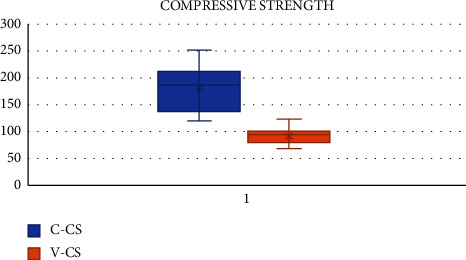
Box plot showing comparison of compressive strength of groups A and B expressed in MPa performed using the Mann–Whitney-*U* test showed a statistically significant difference (*p* < 0.05), where group A shows higher compressive strength than group B (180.84 > 90.96).

The CS of group A was significantly higher than group B (*p*  <  0.05). SPSS 22.0 (SPSS Inc., Chicago, IL) software was used to analyse the data. The level of significance was set at *p* < 0.05. The Mann–Whitney-*U* test analysis conducted between the two test groups regarding the CS reported a statistically significant difference (*p* < 0.05), wherein group A showed better CS than group B (180.84 > 40.84) (Table 1 and Figure 3).

## 4. Discussion

Resin composites, conventional and modified glass ionomers, and compomers are commonly used restorative materials for both permanent and primary teeth. But the choice of the restorative material varies from permanent to primary teeth. Although composites are considered as best in terms of esthetics and mechanical properties, they are not used in all pediatric patients even though they are indicated. The use of composites for restoration of especially voluminous cavities requires an elaborate application technique that results in longer chair side time which is not suitable for children with a short attention span. In primary teeth, the volume of pulp is comparatively larger and the pulp lies closer to dentin; hence, children manifest with higher postoperative sensitivity due to polymerization shrinkage in composite restorations [14]. Bulk-filled, self-adhesive, and rapidly cured restorative materials such as RMGIC are therefore preferred. They offer easy and effective solutions for the practice of pediatric operative dentistry [15].

Cention N is a subgroup of the composite resin with an alkasite-based filler. However, it is available in powder and liquid forms. The restorative material obtained from the mixing of powder and liquid as per manufacturer's recommendation can polymerise itself or can be light activated to polymerise [16]. Considering the lesser chair side time available for restorations of pediatric patients, Cention N can be a better choice over composite. Both Cention N and RMGIC can be bulk filled and self-cured; thus, a comparison was intended between these two materials in this study. On the one hand, RMGIC is a time-tested material with good clinical performance, and on the other hand, Cention N claims better anticariogenic properties. On the one hand, RMGIC is a time-tested material with good clinical performance, and on the other hand, Cention N claims better anticariogenic and mechanical properties. Previous in vitro studies comparing Cention N to resin modified GIC evaluated parameters such as fluoride releasing ability, antibacterial property, flexural strength, and microleakage [5, 16–19]. Our study compared the compressive strength, shear bond strength, and flexural strength of Cention N and RMGIC.

The clinical success of a bulk-filled restorative material is determined by its ability to adhere to the dentinal surface and withstand the various dislodging forces that act within the oral cavity [11, 14]. Mechanical properties of a given restorative material including shear bond strength, compressive strength, and flexural strength play a major role with respect to the long-term survival of bulk-filled restorations in posterior teeth involving occlusal and occlusoproximal cavities. Hence, in our study, we evaluated and compared the CS, FS, and SBS of CN with RMGIC.

SBS is the ability of two materials to withstand sliding or twisting forces applied at their junction. In posterior teeth, high shearing forces are exerted during mastication, which will result in the restorative material being debonded from the tooth surface [12, 18–20].

The results of SBS in our study showed a mean shear bond strength of 7.894 ± 4.76 MPa and 18.89 ± 5.82 MPa in group A (CN) and group B (RMGIC), respectively. RMGIC showed statistically significantly higher mean SBS when compared to CN. The mean SBS values obtained in group A (CN) in our study correspond to the values obtained in other studies [14, 19]. These studies compared Cention to type IX and type II GIC. In our study, Cention N was directly bonded to tooth, whereas preconditioning of the tooth was performed in group B (RMGIC) as recommended by the manufacturer; this could influence the SBS values. Hence, SBS of Cention N is lower than that of RMGIC as seen in the results of our study.

Mazumdar et al. conducted a study evaluating the SBS of Cention N to enamel and dentin with and without the use of an etchant. The SBS of CN to nonetched enamel was 1.46 MPa, whereas to nonetched dentin, it was 1.05 MPa [21]. On the other hand, the SBS of CN to etched enamel and dentin was 1.92 MPa and 1.43 MPa, respectively. These observations reveal that the SBS of Cention N will be higher on the pretreated surface than on the untreated tooth surface.

Similar findings were observed by Francois et al., who concluded that SBS was higher when CN was bonded with a universal adhesive system than when CN was bonded directly to the tooth surface [22].

In order to increase the SBS of Cention N, the manufacturer (Ivoclar) has now launched a new product called Cention Forte to address this drawback. They now offer a matching primer, resulting in a completely coordinated system consisting of Cention Forte (CF) and Cention Primer (CP) for basic dental fillings. The two-component CP was designed specifically for use with CF. The self-etching and self-curing primers offer an excellent foundation for enhancing the bond strength of the material [8].

Assessment of bond failure can give an indication of the nature of the bond between the restorative material and tooth structure. Adhesive failures refer to the disruption of bonds between the molecules or atoms of two different types of materials, while cohesive failures refer to the disruption of bonds between molecules or atoms of the same species [13]. The stereomicroscope and SEM were used at various magnifications in this investigation to assess the forms of failure. In our study, 9 samples of CN had adhesive failures, whereas 2 had mixed failures. In the case of RMGIC, 8 samples had adhesive failures, 2 had mixed failure, and 1 had cohesive failure.

The FS is used to determine the strength of the material and the degree of distortion that can be expected under bending forces. In clinical conditions, flexural forces are generated and materials must be able to tolerate repeated flexing, bending, and twisting forces [2, 5]. The mean FS in MPa obtained for CN in our study was 156.46 ± 16.01, and it was statistically significantly higher than the mean FS obtained for RMGIC which was 90.69 ± 15.57. The results reveal that the comparison of FS of Cention N is higher than RMGIC, which is in agreement with other studies comparing Cention N with composites, RMGIC, and type IX GIC [5, 9, 22].

The CS of restorative materials determines the ability of the material to withstand intraoral compressive and tensile forces generated during function and parafunction, i.e., during mastication [10, 23]. As observed in our study, the CS in MPa, for CN, was 180.84 ± 40.84, whereas for RMGIC, it was 90.96 ± 14.56. Thus, CN had statistically significantly higher CS than RMGIC closely similar to the study conducted by Verma et al. and Kaur et al. [9, 14].

Cention consists of a combination of UDMA, DCP, an aromatic aliphatic-UDMA, and PEG-400 DMA that forms cross-links during polymerization resulting in strong mechanical properties. UDMA is the main component of the monomer matrix which exhibits moderate viscosity and yields strong mechanical properties. The inorganic filler barium-aluminum-silicate glass imparts strength to the material [5]. This explains the better mechanical properties of the material.

Based on the results of the current study, the null hypothesis stated as “there is no difference in compressive strength and flexural strength between Cention N and RMGIC” was rejected. The results of this study are summarised as Cention N showed higher flexural and compressive strengths compared to RMGIC, while RMGIC showed higher shear bond strength compared to Cention N.

The clinical performance of any restoration varies significantly from in vitro conditions as exact replication of intraoral conditions and stress is nearly impossible. Hence, the limitations of the current in vitro study are that the clinical performance of this material may vary in in vivo conditions. It is therefore recommended that further research studies evaluating the clinical performance of this material in in vivo conditions are required. The Cention N and Cention Forte are both self- and dual-cured materials. Further studies are required to justify the use of either type of curing with specific indications for the same.

## 5. Conclusion

From the findings of this study, CN can be considered a superior material to RMGIC, especially in terms of compressive and flexural strengths. The shear bond strength of CN was not better than the RMGIC. This can be improved with the addition of a primer-bonding system which the manufacturers have introduced with CF. Thus, CN can be considered to be a satisfactory bulk-filled, self-cured, or bulk-cured restorative material for primary posterior teeth.

## Figures and Tables

**Table 1:** Intergroup comparison using the Mann–Whitney-*U* test.

Groups (*N* = 11)	SBS	Flexural strength	Compressive strength
Group A—Cention N	7.894 ± 4.76	156.46 ± 16.01	180.84 ± 40.83
Group B—RMGIC	18.89 ± 5.82	90.69 ± 15.57	90.96 ± 14.56
*P* value	0.0008^*∗*^	0.0008^*∗*^	0.0001^*∗*^

^*∗*^ *P* < 0.05 is statistically significant, reporting a significant difference between the groups for shear bond strength, flexural strength, and compressive strength.

## Data Availability

The data obtained from this study are available from the corresponding author upon request.
